# Mature human induced pluripotent stem cell-derived cardiomyocytes promote angiogenesis through alpha-B crystallin

**DOI:** 10.1186/s13287-023-03468-4

**Published:** 2023-09-07

**Authors:** Yuki Tanaka, Shin Kadota, Jian Zhao, Hideki Kobayashi, Satomi Okano, Masaki Izumi, Yusuke Honda, Hajime Ichimura, Naoko Shiba, Takeshi Uemura, Yuko Wada, Shinichiro Chuma, Tsutomu Nakada, Shugo Tohyama, Keiichi Fukuda, Mitsuhiko Yamada, Tatsuichiro Seto, Koichiro Kuwahara, Yuji Shiba

**Affiliations:** 1 Department of Regenerative Science and Medicine, Shinshu University School of Medicine, 3-1-1 Asahi, Matsumoto, 390-8621 Japan; 2 Institute for Biomedical Sciences, Shinshu University, Matsumoto, 390-8621 Japan; 3 Division of Cardiovascular Surgery, Department of Surgery, Shinshu University School of Medicine, Matsumoto, 390-8621 Japan; 4 Department of Cardiovascular Medicine, Shinshu University School of Medicine, Matsumoto, 390-8621 Japan; 5 Department of Pediatrics, Shinshu University School of Medicine, Matsumoto, 390-8621 Japan; 6 Division of Gene Research, Research Center for Advanced Science and Technology, Shinshu University, Matsumoto, 390-8621 Japan; 7 Department of Regeneration Science and Engineering, Institute for Life and Medical Sciences, Kyoto University, Kyoto, 606-8507 Japan; 8 Division of Instrumental Analysis, Research Center for Advanced Science and Technology, Shinshu University, Matsumoto, 390-8621 Japan; 9 Department of Cardiology, Keio University School of Medicine, Tokyo, 160-8582 Japan; 10 Department of Molecular Pharmacology, Shinshu University School of Medicine, Matsumoto, 390-8621 Japan; 11 Present Address: Department of Physical Therapy, Faculty of Health Sciences, Iryo Sosei University, Iwaki, 970-8551 Japan; 12 Present Address: Division of Diabetes, Endocrinology and Metabolism, Department of Internal Medicine, Shinshu University School of Medicine, Matsumoto, 390-8621 Japan

**Keywords:** Human induced pluripotent stem cell-derived cardiomyocytes, Engraftment, Cell transplantation, Angiogenesis, Maturation, CRYAB

## Abstract

**Background:**

Human induced pluripotent stem cell-derived cardiomyocytes (hiPSC-CMs) can be used to treat heart diseases; however, the optimal maturity of hiPSC-CMs for effective regenerative medicine remains unclear. We aimed to investigate the benefits of long-term cultured mature hiPSC-CMs in injured rat hearts.

**Methods:**

Cardiomyocytes were differentiated from hiPSCs via monolayer culturing, and the cells were harvested on day 28 or 56 (D28-CMs or D56-CMs, respectively) after differentiation. We transplanted D28-CMs or D56-CMs into the hearts of rat myocardial infarction models and examined cell retention and engraftment via in vivo bioluminescence imaging and histological analysis. We performed transcriptomic sequencing analysis to elucidate the genetic profiles before and after hiPSC-CM transplantation.

**Results:**

Upregulated expression of mature sarcomere genes in vitro was observed in D56-CMs compared with D28-CMs. In vivo bioluminescence imaging studies revealed increased bioluminescence intensity of D56-CMs at 8 and 12 weeks post-transplantation. Histological and immunohistochemical analyses showed that D56-CMs promoted engraftment and maturation in the graft area at 12 weeks post-transplantation. Notably, D56-CMs consistently promoted microvessel formation in the graft area from 1 to 12 weeks post-transplantation. Transcriptomic sequencing analysis revealed that compared with the engrafted D28-CMs, the engrafted D56-CMs enriched genes related to blood vessel regulation at 12 weeks post-transplantation. As shown by transcriptomic and western blot analyses, the expression of a small heat shock protein, alpha-B crystallin (CRYAB), was significantly upregulated in D56-CMs compared with D28-CMs. Endothelial cell migration was inhibited by small interfering RNA-mediated knockdown of CRYAB when co-cultured with D56-CMs in vitro. Furthermore, CRYAB overexpression enhanced angiogenesis in the D28-CM grafts at 4 weeks post-transplantation.

**Conclusions:**

Long-term cultured mature hiPSC-CMs promoted engraftment, maturation and angiogenesis post-transplantation in infarcted rat hearts. CRYAB, which was highly expressed in D56-CMs, was identified as an angiogenic factor from mature hiPSC-CMs. This study revealed the benefits of long-term culture, which may enhance the therapeutic potential of hiPSC-CMs.

**Supplementary Information:**

The online version contains supplementary material available at 10.1186/s13287-023-03468-4.

## Introduction

Cardiac regenerative therapy using human pluripotent stem cell-derived cardiomyocytes (hPSC-CMs) is expected to be a next-generation therapy for failing hearts because of the unlimited cardiogenic potential of hPSC-CMs. Accumulating evidence has revealed that PSC-CMs can heal injured hearts through not only indirect paracrine effects but also direct remuscularization [1–5]. Although clinical studies have been initiated in several countries, hPSC-CM treatments still have some critical limitations, including poor engraftment of the transplanted CMs. One potential approach to increase the engraftment efficiency is the promotion of cell cycle activity in hPSC-CMs [6, 7], but this strategy may enhance the risk of tumorigenicity. In general, the maturity of hPSC-CMs is inversely correlated with their proliferative capacity [8, 9]. Thus, one potential advantage of transplanting immature cardiac progenitor cells is their higher proliferative capacity; however, hPSC-CMs younger than 10 days old were shown to be too immature to engraft. For example, 8-day-old hPSC-CMs demonstrated lower engraftment efficiency than 20-day-old hPSC-CMs post-transplantation in mouse hearts [10]. Moreover, 3-day-old hPSC-derived cardiovascular progenitor cells could not engraft or survive in infarcted non-human primate (NHP) hearts [11], whereas 20-day-old PSC-CMs clearly achieved remuscularization in NHP hearts [1–3]. These data suggest that proliferation is not the only factor contributing to hPSC-CM engraftment. Furthermore, one disadvantage of transplanting immature PSC-CMs is arrhythmogenesis after engraftment [1–3, 9, 12, 13], which is another limitation of this treatment; therefore, at least some promotion of cell maturation is necessary before transplantation.

Several approaches, including mechanical stimulation combined with tissue engineering [14], chemical treatment [15, 16] and in vivo environment [17, 18], have been reported to promote hPSC-CM maturation. Long-term culture is the most straightforward method employed to promote hPSC-CM maturation and results in proper sarcomere organization, increased cell size, enhanced contractile performance and improved calcium handling and metabolic output [19, 20]. Recently, artificially matured hPSC-CMs were transplanted into injured rat [21] or guinea pig hearts [22], but it remained unclear whether mature hPSC-CMs are superior in terms of engraftment efficiency. Moreover, the optimal maturation marker of hPSC-CMs for classifying their regenerative potential is unknown. The mature isoform predominance of multiple sarcomere-related genes has been reported to be an indicator of mature CMs in several reviews [9, 23]. In a proteomics study, a 2-month prolonged culture of hPSC-CMs showed isoform switching from alpha myosin heavy chain (αMHC; *MYH6*) to beta myosin heavy chain (βMHC; *MYH7*), from myosin light chain 2 atrial isoform (MLC-2a; *MYL7*) to ventricular isoform (MLC-2v; *MYL2*) and from slow skeletal troponin I (ssTnI; *TNNI1*) to cardiac troponin I (cTnI; *TNNI3*) compared to the results in a one-month culture [24]. Thus, we prepared 56-day-old hiPSC-CMs as mature CMs and 28-day-old hiPSC-CMs as controls to compare their engraftment efficiency and maturity in a rat model of myocardial infarction. We also investigated the mechanism of the engraftment of mature hiPSC-CMs, leading to the identification of a pleiotropic maturation marker. These findings will pave the way for future clinical applications of cardiac regeneration.

## Methods

### Cardiomyocyte differentiation

**Fig. 1:**
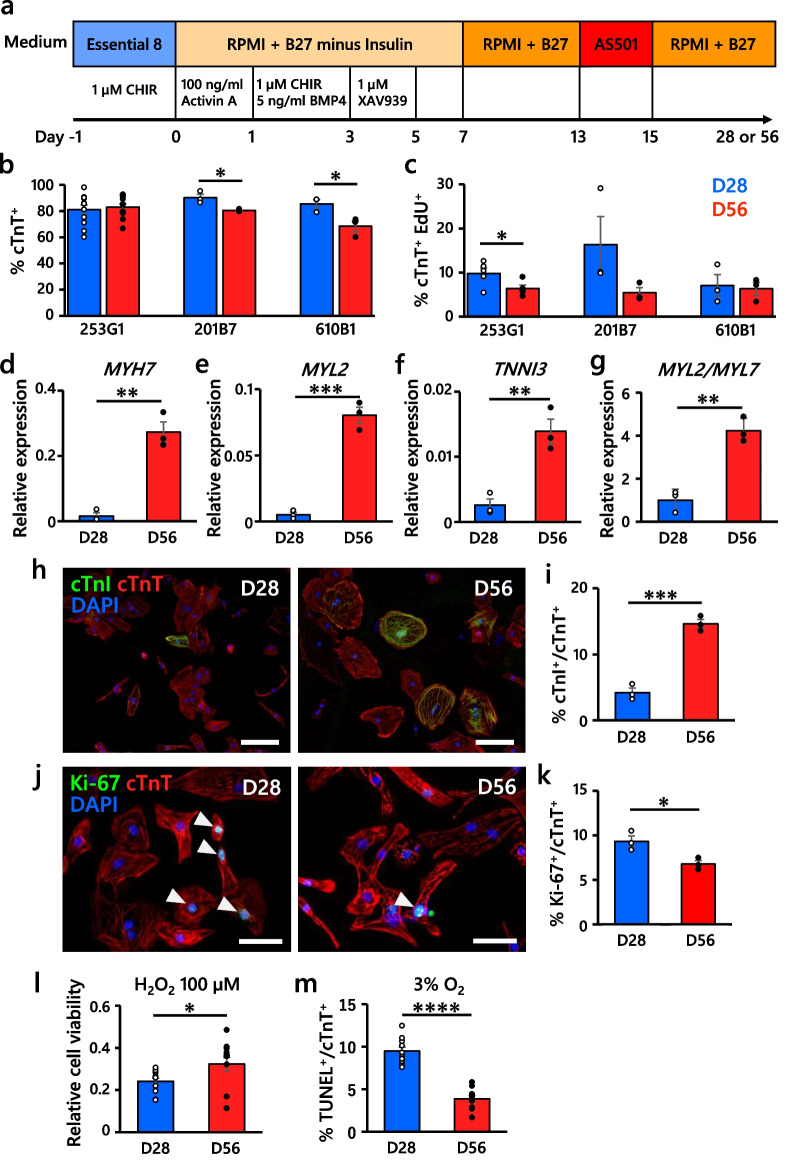
Characterization of hiPSC-CMs. **a** Schematic representation of the monolayer-based differentiation protocol for hiPSC-CMs harvested and cryopreserved on day 28 (D28) or day 56 (D56), shown in blue or red bars below, respectively. **b** Percentage of cTnT^+^ cells. *n* = 11, 3 and 3 in 253G1, 201B7 and 610B1 hiPSC-derived cells, respectively. **c** Percentage of cTnT^+^ and EdU^+^ cells. *n* = 5, 3 and 3 in 253G1, 201B7 and 610B1 hiPSC-derived cells, respectively. **d**–**f** qRT-PCR analysis of the relative mRNA expression of mature sarcomere genes in 253G1-derived CMs. The value for an adult heart sample was set to 1 as a reference. *n* = 3 each. **g** Ratio of mature/immature sarcomere gene expression in 253G1-derived CMs. *n* = 3 each. **h** Representative images of mature CMs derived from 253G1 hiPSC-CMs detected by cTnI and cTnT immunostaining. Scale bars, 100 µm. **i** Percentage of mature CMs derived from 253G1. *n* = 3 each. **j** Representative images of proliferating 253G1 hiPSC-CMs (white arrowheads) detected by Ki-67 and cTnT immunostaining. Scale bars, 50 µm. **k** Percentage of 253G1-derived proliferating CMs. *n* = 3 each. **l** Quantification of 253G1-derived CM viability after treatment with H_2_O_2_ (100 µM) for 2 h. Each value was compared with that of the H_2_O_2_ non-treated group. *n* = 13 per group. **m** Quantification of 253G1-derived apoptotic CMs after hypoxia (3% O_2_ 24 h)–reoxygenation. *n* = 10 each. All data are presented as the mean ± SEM, and the *P* values were determined with unpaired t tests, except **l** with the Mann–Whitney U test (*P < 0.05, **P < 0.01, ***P < 0.001, ****P < 0.0001)

hiPSC-CMs were differentiated from 253G1, 201B7 and 610B1 hiPSCs (RIKEN BioResource Center, Tsukuba, Japan) by a previously reported cardiac differentiation protocol [17, 25] with some modifications, as illustrated in Fig. 1a. Undifferentiated hiPSCs were cultured on Matrigel (Corning, NY, USA)-coated dishes in Essential 8 medium (Thermo Fisher Scientific, Waltham, MA, USA). Directed differentiation of hiPSC-CMs was performed on high-density monolayers with 100 ng/mL activin A (R&D Systems, Minneapolis, MN, USA), 5 ng/mL bone morphogenetic protein 4 (R&D Systems), 1 µM CHIR99021 (Sigma-Aldrich, St. Louis, MO, USA) and 1 µM XAV939 (Sigma-Aldrich). From day 7, the cells were incubated with RPMI-1640 plus B27 supplement medium (Thermo Fisher Scientific), except on days 13–15, the culture medium was replaced with glucose- and glutamine-free medium with 4 mM lactate (StemFit medium AS501, Ajinomoto, Tokyo, Japan) [26–28].

### Generation of the fluorescent and luminescent reporter hiPSC lines

A transgene for the constitutive expression of GCaMP3 was inserted into the adeno-associated virus integration site 1 (*AAVS1*) locus in 253G1 hiPSCs via zinc-finger nuclease-mediated transgenesis as previously reported [29, 30]. Venus-Akaluc-expressing hiPSCs were generated via clustered regularly interspaced short palindromic repeats (CRISPR)-associated nuclease 9 (CRISPR/Cas9)-mediated transgenesis. In brief, a donor plasmid using the pSF-CAG-Ub-Puro (Sigma-Aldrich; OGS600) cloning vector was generated, consisting of approximately 800-bp-long homology arms flanking the *AAVS1* sgRNA (ccaatcctgtccctagtggcccc) cut site surrounded by an 8.4-kb insert with two elements, namely a cassette bearing the CAG promoter that drives Venus-Akaluc expression and a second cassette for Ubc-driven expression of puromycin resistance gene surrounded by LoxP sites, as illustrated in Additional file 1: Fig. S1a. We obtained the Venus-Akaluc sequence from the RIKEN BioResource Center (pcDNA3 Venus-Akaluc; RDB15781). The plasmids expressing sgRNA and Cas9 were constructed using the Guide-it CRISPR/Cas9 system (Takara Bio, Shiga, Japan; 632601) according to the manufacturer’s protocols. A day prior to electroporation, hiPSCs were treated with 10 µM valproic acid for 24 h. Venus-Akaluc donor plasmid (6 µg), Cas9/sgRNA plasmid (3 µg) and 1 µg RAD51-expressing plasmids [31] were co-electroporated by Nucleofector 2b (Lonza, Basel, Switzerland) with the B-16 program into 1 × 10^6^ hiPSCs. Two days after electroporation, transfected cells were selected with 1 µg/mL puromycin for 4 days. After expansion, PCR genotyping was performed to determine whether the clones were correctly targeted. Then, the pCAG-iCre plasmid (10 µg; Addgene, Watertown, MA, USA; 89573) was electroporated into the correctly targeted clones to remove the selection cassette (Additional file 1: Fig. S1b, c). Luc2 (Promega, Madison, WI, USA; E6651)-expressing hiPSCs were generated in the same manner as Venus-Akaluc-expressing hiPSCs. Genomic DNA was isolated using the DNeasy Blood and Tissue Kit (Qiagen, Venlo, Netherlands) according to the standard protocol. The region around the cut site was amplified with PrimeSTAR GXL DNA polymerase (Takara Bio), and the primers are indicated in Additional file 1: Fig. S1a. PCR products were visualized on a 1% agarose gel. The primer sequences are provided in Additional file 1: Table S1.

### Cell preparation for transplantation

The hiPSC-CMs used in this study were cryopreserved on days 28 and 56 of differentiation (D28-CMs and D56-CMs, respectively) and thawed immediately prior to cell injection as per our previously described protocol [2, 30]. A day prior to cryopreservation, the cells were subjected to heat shock at 43 °C for 30 min. Prior to enzymatic dispersion, 100 ng/mL insulin-like growth factor 1 (IGF1, PeproTech, Cranbury, NJ, USA), 200 nM cyclosporine A (Novartis, Basel, Switzerland) and 10 µM Y-27632 (FUJIFILM Wako Pure Chemical, Osaka, Japan) were added to the culture medium overnight, and the cells were dispersed by incubating them with TrypLE Select or 0.05% Trypsin EDTA (Thermo Fisher Scientific). To visualize proliferating cells, hiPSC-CMs were labeled with 10 μM EdU (Click-iT EdU Imaging Kits, C10337, Thermo Fisher Scientific) 1 day prior to cell harvest. hiPSC-CMs were resuspended in CELLBANKER 1 plus reagent (Takara Bio, CB023) and frozen in cryovials in a controlled-rate freezer at − 80 °C before storage in a freezer at − 150 °C. For thawing, the cryopreserved cells were washed with RPMI-1640 plus B27 supplement medium and resuspended in an RPMI-based prosurvival cocktail containing 50% growth factor-reduced Matrigel, 100 mM ZVAD [benzyloxycarbonyl-Val-Ala-Asp(O-methyl)-fluoromethyl ketone, Calbiochem, San Diego, CA, USA], 50 nM Bcl-XL BH4 (cell-permeant TAT peptide, Calbiochem), 200 nM cyclosporine A (Novartis), 100 ng/mL IGF1 (PeproTech) and 50 µM pinacidil (Sigma-Aldrich).

### Animal surgery

For all in vivo studies, group sizes were estimated based on power analyses using previous study variance [25, 30, 32]. While no formal methods of randomization were used, the animals were randomly selected by a surgeon who was blinded to the treatments used. Male athymic rats (9–11 weeks old) (F344/NJcl-rnu/rnu, CLEA Japan, Tokyo) were injected with D28-CMs (*n* = 27), D56-CMs (*n* = 27), CRYAB-OE D28-CMs (*n* = 4), tdTomato-OE D28-CMs (*n* = 4), undifferentiated hiPSCs (*n* = 1) or phosphate-buffered saline (PBS) control (*n* = 5). After transplanting D28- or D56-CMs, 5, 5, 5 and 12 rats were euthanized at 1, 4, 8 and 12 weeks, respectively. Echocardiography was performed for 5 animals per group, in vivo bioluminescence imaging was performed for 5–6 animals per group, and RNA sequencing (RNA-seq) analysis was performed for 2 animals per group, which were euthanized at 12 weeks post-transplantation.

#### Drugs used in the animal study

Mixed anesthesia with medetomidine–midazolam–butorphanol (MMB) was prepared with 0.15 mg/kg medetomidine hydrochloride (Dolben, Kyoritsu Seiyaku, Tokyo, Japan), 2.0 mg/kg midazolam (Dormicum, Maruishi Seiyaku, Osaka, Japan) and 2.5 mg/kg butorphanol tartrate (Vetorphale, Meiji Seika Pharma, Tokyo, Japan) and diluted with saline (Otsuka Pharma, Tokyo, Japan) to administer a dose of 0.5 mL/100 g body weight. Atipamezole hydrochloride (Atipame, Kyoritsu Seiyaku), an antagonist of medetomidine, was administered at a dose of 0.15 mg/kg body weight to promote recovery from anesthesia.

#### Myocardial infarction model

Rats were anesthetized via a single subcutaneous administration of MMB, followed by intubation and inhalation of 2–3% sevoflurane (Mylan, Canonsburg, PA, USA) using a mechanical ventilator. Surgery was performed on a warming pad to maintain body temperature. For the induction of myocardial infarction, the left anterior chest was incised, the intercostal space was opened with a thoracotomy device to expose the heart, and the middle part of the left anterior descending artery was ligated using 6–0 braided silk (Natsume Seisakusho, Tokyo, Japan). The chest was then closed using 4–0 braided silk (Natsume Seisakusho). Meloxicam (0.2 mg/kg; Metacam, Boehringer Ingelheim, Tokyo, Japan) diluted in saline (Otsuka Pharma) was subcutaneously administered at a dose of 0.05 mL/kg body weight. After intramuscular injection of Atipame, the rats were extubated upon the recovery of spontaneous breathing.

#### Cell transplantation

Based on our previous studies showing the efficient engraftment of hPSC-CMs [25, 30], on day 7 after inducing myocardial infarction, cryopreserved hiPSC-CMs (2 × 10^7^) were thawed and suspended in a prosurvival cocktail containing Matrigel to adjust the volume to 70 µL per animal. Cell suspension or PBS (control) was directly injected at two sites on the anterior wall of the rat heart using a 29-gauge injection needle.

For the comparative study of Luc2- and Akaluc-expressing hiPSCs, undifferentiated hiPSCs (5 × 10^5^) with Matrigel were transplanted subcutaneously into the back of the recipient rat. MMB anesthetics, antagonists and analgesics used during cell transplantation were the same as those used in surgery to induce myocardial infarction.

### Histology and immunohistochemistry

At the end of the animal study, rats were euthanized via intraperitoneal administration of MMB (5 times the induction dose) followed by injection of potassium chloride from the inferior vena cava. Rat hearts were harvested, washed with PBS, sliced at 2 mm thickness and fixed overnight in 4% paraformaldehyde. After embedding, paraffin blocks were sectioned at 5 μm thickness and stained as mentioned below. All sections were routinely stained with hematoxylin–eosin (HE) and picrosirius red to determine the scar region. For immunohistochemistry, slides were deparaffinized and subjected to heat-mediated antigen retrieval with citric acid buffer (pH 6) or Tris–EDTA buffer (pH 9) for 15 min and blocked with 1.5% normal goat or donkey serum (Jackson ImmunoResearch, West Grove, PA, USA) for 1 h at room temperature, followed by overnight incubation with primary antibodies at 4 °C.

For bright-field studies, the sections were incubated with a horseradish peroxidase (HRP)–polymer secondary antibody at room temperature for 1 h on the consecutive day of primary antibody staining, and chromogenic detection was performed with diaminobenzidine (DAB Substrate Kit, SK-4100, Vector, Newark, CA, USA) staining, followed by counterstaining with hematoxylin. Bright-field images were captured using a NanoZoomer-RS (Hamamatsu Photonics, Shizuoka, Japan) and further analyzed with NDP.view 2.6.13 (Hamamatsu Photonics) or ImageJ (NIH, USA) software.

For fluorescence studies, the sections were incubated with fluorescent secondary antibodies at room temperature for 1 h, followed by counterstaining with 4′,6-diamidino-2-phenylindole (DAPI), if not stained with Ku80. Images were captured with a z-stack using a BZ-X710 inverted microscope (Keyence, Osaka, Japan) and quantified using ImageJ software. The antibodies used in this experiment are listed in Additional file 1: Table S2. For the terminal deoxynucleotidyl transferase dUTP nick end labeling (TUNEL) assay, we used an In Situ Cell Death Detection Kit, TMR red (Sigma-Aldrich, 12156792910), according to the manufacturer’s protocol.

Engrafted hiPSC-CMs were identified with green fluorescent protein (GFP), βMHC or human-specific antibodies, including nucleolin. GFP-positive areas were measured for the quantification of graft size. Sarcomere length, cell sectional area and microvessel density in the graft area were measured by alpha actinin, wheat germ agglutinin and CD31 staining, respectively. For the measurement of sarcomere length, we selected myofibrils with ten continuous, well-recognized alpha-actinin-positive bands and divided the length by ten.

### Immunocytochemistry

Cryopreserved D28- or D56-CMs were thawed and seeded at 2 × 10^5^ cells per well into 12-well plates coated with Matrigel. Seven days after seeding, the cells were fixed in 2% paraformaldehyde for 10 min, blocked with 1.5% normal goat serum (Jackson ImmunoResearch) with 0.5% Triton X-100 for 1 h and incubated overnight with primary antibodies at 4 °C. The next day, the cells were incubated with secondary antibodies at room temperature for 1 h, followed by DAPI counterstaining for 10 min. Images were acquired for analysis with a microscope (Keyence, BZ-X710) and quantified using ImageJ software.

### Cell viability assay

Cell viability was assessed using a Cell Counting Kit (CCK-8, Dojindo, Kumamoto, Japan). Cells were seeded in 96-well plates at 5 × 10^4^ cells per well and incubated for 4 days in RPMI-1640 plus B27 supplement medium. Five days after seeding, the cells were exposed to 100 µM H_2_O_2_ or DMEM (FUJIFILM Wako Pure Chemical), and 10 μL of CCK-8 stock solution was added to each well and incubated at 37 °C for 2 h. The absorbance at 450 nm was measured with a microplate reader (SpectraMax iD5, Molecular Devices, San Jose, CA, USA).

### Echocardiography

Echocardiography was performed 5 days after inducing myocardial infarction (pre-transplantation) and at 4, 8 and 12 weeks post-transplantation. The animals were anesthetized, and the left ventricular end-diastolic dimension (LVEDd), left ventricular end-systolic dimension (LVEDs) and heart rate were measured via transthoracic echocardiography (Vevo2100; FUJIFILM VisualSonics, Toronto, ON, Canada) using a 30-MHz transducer (MX400; Primetech, Tokyo, Japan). The left ventricular end-diastolic volume (LVEDV), left ventricular end-systolic volume (LVESV) and left ventricular ejection fraction (LVEF) were measured using the Teichholz method. Fractional shortening (FS) was calculated using the following equation: FS (%) = 100 × [(LVEDd−LVEDs)/LVEDd]. LVEF was calculated using the following formula: LVEF (%) = 100 × [(LVEDV−LVESV)/LVEDV]. All measurements were taken over three consecutive cardiac cycles, and their average values were calculated. An operator who was blinded to the study groups performed all the measurements.

### Bioluminescence imaging (BLI)

The luminescent substrate AkaLumine-HCl (TokeOni, Sigma-Aldrich, 808350) was diluted to 10 mM with saline and stored at − 80 °C. BLI was performed using an imaging system (NightOWL II LB983, Berthold, Bad Wildbad, Germany) and analyzed using Indigo2 software.

For in vitro BLI, varying quantities (0, 1 × 10^3^, 5 × 10^3^, 1 × 10^4^, 5 × 10^4^ and 1 × 10^5^) of 28- and 56-day-old Akaluc-expressing hiPSC-CMs were seeded on a 96-well plate. On the following day, cell images were captured with 4 × 4 binning and 1-min exposure time immediately after treating the cells with 500 µM TokeOni (100 µL/well).

For in vivo BLI, rats were anesthetized, and TokeOni (20 nmol/g) was administered intravenously into the rats. Rat images were captured with 8 × 8 binning and 2-min exposure time on days 2, 7, 14, 28, 56 and 84 after hiPSC-CM transplantation and on day 1 after hiPSC transplantation.

### Flow cytometry

CM purity was determined by analyzing cardiac troponin T (cTnT)-positive cells using flow cytometry. The cells were fixed with 4% paraformaldehyde and incubated with mouse monoclonal cTnT antibodies (Thermo Fisher Scientific, clone 13–11; 1:100) or mouse immunoglobulin G1 kappa isotype control (BioLegend, San Diego, CA, USA; clone MG1-45; 1:100), followed by incubation with Alexa Fluor 647-conjugated goat anti-mouse secondary antibodies (Thermo Fisher Scientific, 1:200). The cell proliferation rate was determined using the Click-iT Plus EdU Pacific Blue Flow Cytometry Assay Kit (Thermo Fisher Scientific, C10636). Fluorescence signals were detected on a BD FACSCanto II system (BD Biosciences, San Jose, CA, USA) and analyzed using FACS DIVA software and FlowJo (BD Biosciences) software.

### Quantitative reverse transcription PCR (qRT-PCR)

First-strand cDNA was synthesized from total RNA using the SuperScript IV First-Strand Synthesis System (Thermo Fisher Scientific). qRT-PCR was performed with Fast SYBR Green Master Mix (Thermo Fisher Scientific) and a QuantStudio 3 real-time PCR system (Thermo Fisher Scientific). The copy number for each transcript was expressed relative to that of glyceraldehyde 3-phosphate dehydrogenase (*GAPDH*), and the samples were run in triplicate. The primer sequences are provided in Additional file 1: Table S1.

### Human angiogenesis proteome profiler array

The relative expression levels of 55 angiogenesis-related proteins in hiPSC-CMs were analyzed using a human angiogenesis proteome profiler array kit (ARY007, R&D Systems, Minneapolis, MN, USA) according to the manufacturer’s protocol. Briefly, cell lysates were prepared, and 125 μg of protein was subjected to a proteome profiler array by mixing the samples with a cocktail of biotinylated antibodies and incubating them on a nitrocellulose membrane spotted with capture antibodies in duplicate. Streptavidin–horse radish peroxidase (HRP) and chemiluminescent detection reagents were used to detect protein antibodies bound to the capture antibody. The mean spot pixel density was quantified using ImageJ software.

### Western blot

Pelleted hiPSC-CMs were lysed using M-PER Mammalian Protein Extraction Reagent (Thermo Fisher Scientific). Protein lysates were separated on Any kD Mini-Protean TGX gels (Bio-Rad, Hercules, CA, USA) and transferred onto polyvinylidene difluoride membranes. The membranes were then blocked with Bullet Blocking One reagent (Nacalai Tesque, Kyoto, Japan). Primary antibody staining was performed at 4 °C overnight, followed by secondary antibody staining for 1 h at room temperature. The primary antibodies used in the experiment were as follows: mouse anti-CRYAB (Enzo Life Sciences, Farmingdale, NY, USA; ADI-SPA-222-D, 1:1,000) and mouse anti-GAPDH (Proteintech, Rosemont, IL, USA; 60,004-1-Ig, 1:10,000). The secondary antibody used in the experiment was goat anti-mouse IgG HRP-conjugated (Abcam, ab97023, 1:30,000). Proteins were visualized with Western BLoT Hyper HRP substrate (Takara Bio) and a ChemiDoc Touch Imaging System (Bio-Rad).

### RNA-seq

For in vitro samples, total RNA was isolated using the RNeasy Mini Kit (Qiagen) according to the manufacturer’s protocol, including DNase treatment. Human fetal heart RNA (Takara Bio, 636583) and human adult heart RNA (Takara Bio, 636532) were purchased. For in vivo samples, rat hearts were harvested, sliced, immediately embedded in an OCT-embedding compound and stored at − 80 °C. Tissues were sectioned at 10 µm thickness using a cryostat (Leica, Wetzlar, Germany), and serial sections were mounted with DAPI to visualize the graft area through green fluorescent protein autofluorescence. The graft areas were captured from unstained unfixed specimens attached to membrane-coated slides (Leica, 11600289) using a laser microdissection system (Leica). Total RNA was extracted from the graft areas in rat heart tissues using the Arcturus PicoPure RNA isolation kit (Thermo Fisher Scientific). cDNA was synthesized using the SMART-Seq v4 ultralow input RNA kit for sequencing (Takara Bio). Library preparations were conducted using the Nextera DNA Library Prep Kit and subjected to sequencing on a NovaSeq 6000 platform (Illumina, San Diego, CA, USA).

RNA-seq reads were trimmed using Trimmomatic (v0.39) with the following parameter: SLIDINGWINDOW: 10:30 [33]. All samples were separated into human and rat reads using Xenome (v1.0.0) [34]. Moreover, reads classified as human were mapped to the hg38 reference using STAR (v2.7.2a) [35], and a gene count matrix was generated using featureCounts (v1.6.4) [36]. Differential expression analysis was performed with the edgeR package using the trimmed mean of M-values normalization method [37]. Principal component analysis (PCA) was performed, and a plot was generated using the PCAtools package (https://github.com/kevinblighe/PCAtools) with transcripts per million normalization. Finally, Gene Ontology (GO) enrichment analysis was performed using the clusterProfiler package [38].

### Human umbilical vein endothelial cell (HUVEC) migration assay

For the migration assay, 24-well Transwell plates with a 6.5-mm-diameter and polycarbonate membrane inserts with an 8-µm pore size (Corning 3422) were used. hiPSC-CMs were seeded at a density of 5 × 10^5^ cells per well and cultured in 500 µL of RPMI-B27 medium with 5 µM Y-27632 in the lower chamber of a 24-well Transwell plate precoated with Matrigel. HUVECs (Lonza C2519A, Lot 18TL075851) were cultured in EBM2 medium with EGM2 supplements (Lonza CC-3162). HUVECs were used for subsequent experiments at passages 3–4. After 24 h of hiPSC-CM seeding, all media were replaced with RPMI-B27 without Y-27632. Two days after the hiPSC-CM medium change, HUVECs (5 × 10^4^ cells/well in 200 µL of RPMI-B27 medium) were seeded in the upper Transwell chambers precoated with Matrigel. After incubation at 37 °C for 22 h, HUVECs on the upper surface of the upper chamber were cleaned with cotton swabs, and HUVECs on the lower surface of the upper chamber were fixed with 4% paraformaldehyde for 15 min and then stained with 0.2% crystal violet solution (Sigma-Aldrich) for 10 min. Stained cells were counted under an inverted microscope (Keyence, BZ-X710).

For siRNA knockdown experiments, predesigned siRNAs targeting the *CRYAB* (hs.Ri.CRYAB.13.2) gene or non-targeting negative control, obtained from TriFECTa RNAi kits (Integrated DNA Technologies, Coralville, IA, USA), were used. A mixture of 50 µL of Opti-MEM reduced-serum medium (Thermo Fisher Scientific), 2.5 µL of TransIT-TKO transfection reagent (Takara Bio, V2154) and 10 nM siRNA was transfected into hiPSC-CMs at 24 h post-seeding in a 24-well Transwell plate. After 24 h of transfection, the media were replaced with fresh RPMI-B27. Co-culture with HUVECs was performed 2–3 days after transfection as described above. siRNA-transfected hiPSC-CMs were lysed for western blot analysis 3 days after transfection as described above.

### HUVEC tube formation assay

Cell culture supernatants of both D28-CMs and D56-CMs were collected just before heat shock (one day before cell preservation) and cryopreserved at -80 °C. Cell culture supernatants of siRNA-treated D56-CMs were collected 3 days after transfection and cryopreserved at -80 °C. Fifty microliters of Geltrex (Thermo Fisher Scientific A1413202) was spread evenly in a 96-well plate, which was then placed in an incubator (37 °C) for 30 min to allow the Geltex to solidify. Then, HUVECs (5,000 cells per well) were seeded on the Geltrex in a total volume of 200 µl of supernatant. After 6 h, tube-like structures were imaged using an inverted microscope (Olympus, Tokyo, Japan; CKX53), and the master segment lengths were quantified using the Angiogenesis Analyzer tool from ImageJ [39].

### Enzyme-linked immunosorbent assay (ELISA)

The Human CRYAB ELISA kit (CUSABIO, Houston, TX, USA; CSB-EL006008HU) was used according to the manufacturer’s protocol. Exosomes were isolated from 2 ml of thawed culture supernatant using the Total Exosome Isolation Reagent (Thermo Fisher Scientific 4478359) and lysed with 200 µL of RIPA Lysis buffer containing protease inhibitor and phosphate inhibitor (ATTO, Tokyo, Japan; WSE-7420). One hundred microliters of culture supernatants or lysed exosomes was added to the wells of the ELISA kit.

### Hypoxia–reoxygenation

Hypoxia and reoxygenation were performed using a BIONIX Hypoxic Cell Culture Kit (Sugiyama-Gen, Tokyo, Japan; nBIONIX-3). D28-CMs and D56-CMs were seeded at 8 × 10^4^ cells/well on a glass-bottom 96-well plate (Greiner Bio-One, Kremsmünster, Austria; 655892) precoated with Matrigel and cultured with RPMI-B27 medium. Three days after seeding, the culture medium was replaced with glucose-free DMEM (FUJIFILM Wako Pure Chemical 042–32255) containing 1% MEM non-essential amino acids (FUJIFILM Wako Pure Chemical 139–15651). Two hours after the medium change, the 96-well plate was placed in a plastic bag together with a deoxidizing agent to achieve a decrease to 3% O_2_ measured by an oxygen meter [40]. After the deoxidizing agent was removed, a sealed plastic bag containing a 96-well plate was incubated at 37 °C for 24 h. After hypoxia, a plastic bag was opened, and samples were reoxygenated and incubated for another 24 h at 37 °C [41]. After hypoxia–reoxygenation, cells were fixed with 4% paraformaldehyde for 10 min at room temperature, permeabilized with 0.25% Triton X-100 and stained with cTnT and subsequently TUNEL as described above. Images were acquired and analyzed using the high-content imaging system Operetta® and Harmony® software (PerkinElmer, Waltham, MA, USA).

### AAV-mediated *CRYAB* overexpression

The complete cDNA of human *CRYAB* was cloned into an AAV6 vector (AAVpro® Helper Free System, Takara Bio) in frame with the tdTomato gene (AAV6-CMV-CRYAB-P2A-tdTomato) through a self-cleaving P2A peptide. As a negative control, an empty vector carrying only tdTomato (AAV6-CMV-tdTomato) was also generated. Viruses were packaged in AAVpro® 293 T Cell (Takara Bio), viral stocks were obtained with an AAVpro® Purification Kit (Takara Bio), and titration of the AAV particles was performed with a qPCR AAV Titer Kit (G931, Applied Biological Materials, Richmond, Canada). D28-CMs were infected with AAV one day after thawing with a multiplicity of infection (MOI) of 1,000. The cells were incubated at 37 °C for 24 h, and then, the medium was replaced with RPMI-1640 plus B27 supplement medium. Infected cells were harvested 5 days after infection for cell transplantation or in vitro analysis. *CRYAB* expression was measured via qRT-PCR.

### Statistical analysis

Statistical significance was determined using an unpaired two-tailed t test, Mann–Whitney U test or one-way analysis of variance (ANOVA) with Tukey’s multiple comparison test. Statistical significance was defined at *P* < 0.05. Data are presented as the mean ± standard error of the mean (SEM). SPSS (version 27) and R (version 4.0.3) were used for statistical analyses.

## Results

### Extended culture enhanced the maturation of hiPSC-CMs in vitro

We differentiated CMs from three hiPSC lines (253G1, 201B7 and 610B1) via a monolayer culture technique, as shown in Fig. 1a. We harvested and cryopreserved hiPSC-CMs on days 28 and 56 after cell differentiation (D28-CMs and D56-CMs, respectively). The mean cardiac purity of the D28-CMs and D56-CMs was 81 ± 4% and 83 ± 3% in 253G1-derived CMs, 90 ± 3% and 80 ± 1% in 201B7-derived CMs, and 85 ± 3% and 68 ± 4% in 610B1-derived CMs, respectively (Fig. 1b). The mean proliferation rate, measured by 5-ethynyl-2ʹ-deoxyuridine (EdU) labeling, significantly decreased from 9.78 ± 1.23% on day 28 to 6.40 ± 0.71% on day 56 in 253G1-hiPSC-CMs (Fig. 1c**,** Additional file 1: Fig. S2a, b). qRT-PCR analysis revealed that extended culture of hiPSC-CMs upregulated the expression of mature sarcomere genes, such as *MYH7, MYL2* and *TNNI3,* as well as the ratio of *MYH7/MYH6, MYL2*/*MYL7* and *TNNI3/TNNI1* (Fig. 1d–g, Additional file 1: Fig. S3a-g). Additionally, the expression of hypertrophy (*NPPA* and *NPPB*)- and calcium handling-related genes (*RYR2* and *ATP2A2*) tended to be upregulated in D56-CMs (Additional file 1: Fig. S3h-k). cTnI protein expression was also upregulated in D56-CMs by immunocytochemistry (Fig. 1h, i, Additional file 1: Fig. S4a-d). Collectively, these analyses revealed that extended culture enhanced the maturation of hiPSC-CMs. While the three hiPSC lines exhibited equivalent maturity and proliferative capacity, we selected the 253G1 hiPSC line for the in vivo transplantation study because of substantial experience in our hands [17, 30]. After replating D28-CMs and D56-CMs derived from 253G1 hiPSCs, we assessed the proliferation and cell viability. The mean proliferative rate detected by Ki-67 immunostaining was significantly higher in the D28-CMs (Fig. 1j, k), while cell viability was higher in the D56-CMs after the administration of 100 µM H_2_O_2_ (Fig. 1l**)**. We also investigated apoptosis after hypoxia–reoxygenation by a TUNEL assay, and there were fewer apoptotic cells among D56-CMs than in D28-CMs (Fig. 1m, Additional file 1: Fig. S4e).

### Establishment of an in vivo brighter graft detection system with Akaluc-expressing hiPSCs

To trace and visualize the engrafted hiPSC derivatives in vivo, researchers have typically used D-luciferin with firefly luciferase (Luc2); however, the low permeability weakens the brightness of bioluminescent signals, especially in the deep organs of animals. AkaLumine with Akaluc, which is a genetically modified and improved D-luciferin with Luc2, allowed the visualization of a small number of engrafted cells in the brain of a freely moving common marmoset [42]; thus, we created Akaluc-expressing hiPSCs using CRISPR/Cas9-mediated gene targeting techniques (Additional file 1: FigureFig. S1a-c). When we transplanted the same amount of undifferentiated Akaluc- or Luc2-expressing hiPSCs into the back of a rat, we detected an obviously brighter signal from the Akaluc-expressing hiPSCs than from the conventional Luc2-expressing hiPSCs after the administration of AkaLumine through in vivo BLI (Additional file 1: Fig. S5a, b).

### D56-CMs increased bioluminescence intensity after transplantation

**Fig. 2:**
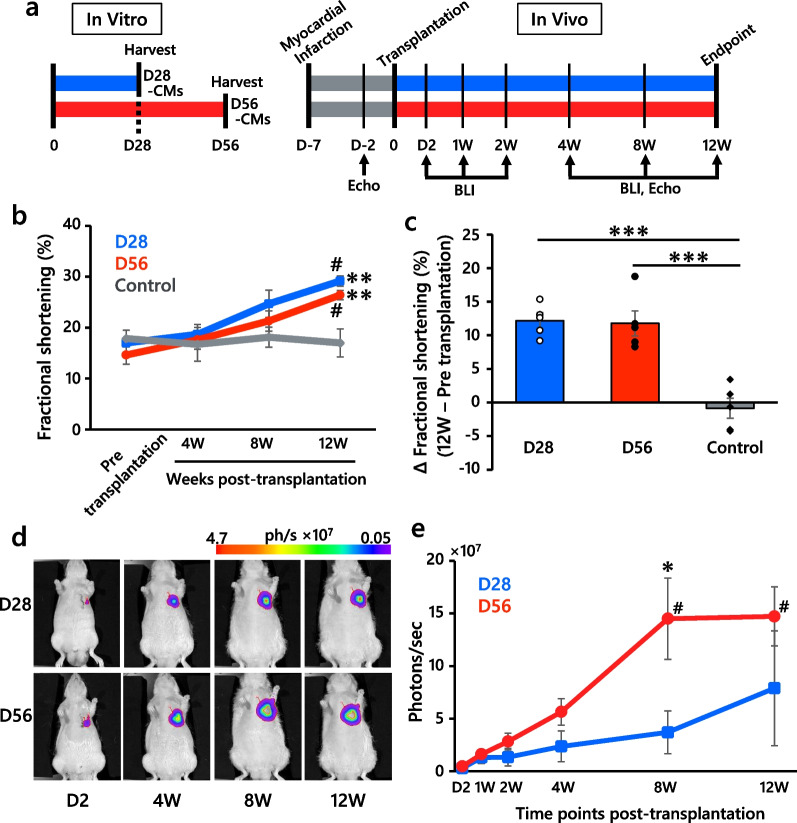
hiPSC-CM transplantation study. **a** Schematic overview of the experimental design. hiPSC-CMs were harvested and cryopreserved on day 28 (D28-CMs) or day 56 (D56-CMs) after differentiation. Myocardial infarction inductions were performed 7 days before (D-7) cell transplantation. Echocardiography (Echo) and BLI were performed over time until 12 weeks after transplantation, when rat hearts were harvested for histological and RNA-seq analyses. **b** Fractional shortening of the left ventricle in echocardiography before and after transplantation in the CM-treated (D28, D56) and PBS-treated control groups. *n* = 5 per group. # *P* < 0.001 versus the value pre-transplantation within the group, **P < 0.01 versus the control group, one-way ANOVA with Tukey’s multiple comparisons test. **c** Change in fractional shortening from pre-transplantation to 12 weeks post-transplantation. *n* = 5 per group. ***P < 0.001, one-way ANOVA with Tukey’s multiple comparisons test. **d** Representative images of bioluminescence signals (ph/s; photons per sec) from transplanted CMs (D28-CMs; top or D56-CMs; bottom) are shown in pseudocolor. From left to right, images at 2 days and 4, 8 and 12 weeks after transplantation (D2, 4 W, 8 W, 12 W) are shown. **e** Time course analysis of bioluminescence intensity after transplantation. D28-CM-treated group, *n* = 5; D56-CM-treated group, *n* = 6. *P < 0.05, unpaired t test, D28 versus D56 at 8 W. #*P* < 0.05, one-way ANOVA with Tukey’s multiple comparison test. 8 W or 12 W versus D2, 1 W, 2 W, 4 W. All data are presented as the mean ± SEM

We transplanted 253G1 hiPSC-derived D28-CMs or D56-CMs by direct cell injection into injured athymic rat hearts 7 days after the induction of myocardial infarction ligating the left anterior descending artery (Fig. 2a). Echocardiography revealed significant improvement in left ventricular contractile function at 12 weeks in both the D28-CM and D56-CM treatment groups compared with the PBS-treated control group (Fig. 2b, c**,** Additional file 1: Fig. S6a-e). There was no significant difference in the bioluminescence intensity between D28-CMs and D56-CMs, and we validated the dose-dependent increase in both groups through in vitro BLI (Additional file 1: Fig. S7a, b). After transplantation, we measured the temporal changes in signal intensity from the hiPSC-CM grafts in rat hearts through in vivo BLI (Fig. 2d, e, Additional file 1: Fig. S7c). In the time series comparison, there was a significant increase in bioluminescence intensity in the D56-CM grafts (*n *= 6, *P* < 0.0001, one-way ANOVA); surprisingly, this increase was not observed in the D28-CM grafts (*n* = 5). While no significant difference in early-stage graft retention was observed between the D28-CM and D56-CM groups, the D56-CM grafts showed brighter signals than the D28-CM grafts at the late time points after 2 weeks. Notably, at 8 weeks post-transplantation, a significantly brighter signal was detected in the D56-CM grafts than in the D28-CM grafts.

### Extended culture promoted hiPSC-CM engraftment and maturation in vivo

**Fig. 3:**
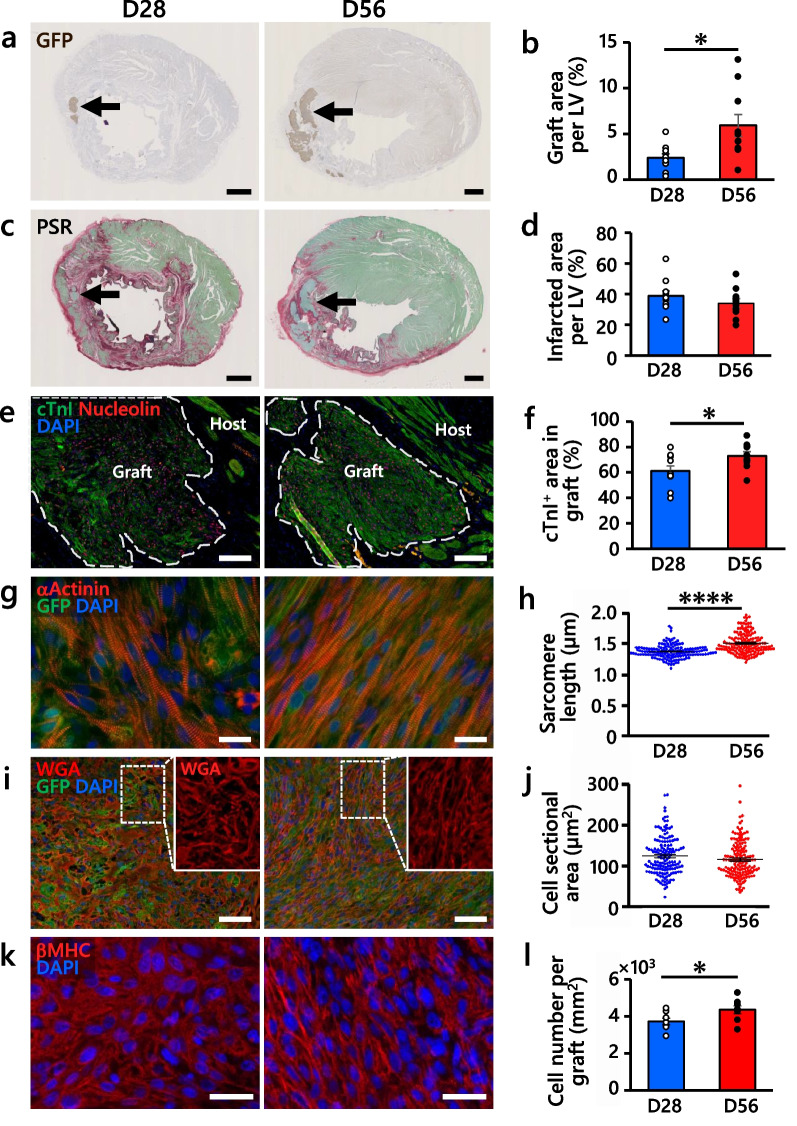
Histological analysis of hiPSC-CM grafts at 12 weeks post-transplantation. The pictures are representative images of D28-CM (left) and D56-CM (right) grafts. The panels show the quantification of the analyzed parameters. **a** Grafts detected by GFP (indicated by arrows) staining. Scale bars, 1 mm. **b** Graft area normalized to the left ventricular (LV) area. *n* = 10 per group. **c** Picrosirius red (PSR) -fast green counterstain shows infarcted (red) and healthy (green) areas. Arrows indicate grafts. Scale bars, 1 mm. **d** Infarcted area normalized to the LV area. *n* = 10 per group. **e** Mature sarcomere structures detected by cTnI (green) in the grafts (white dotted lines). Human nuclear antigen (HuNu); nucleolin, red. Scale bars, 100 µm. **f** Percentage of cTnI^+^ area in the grafts. *n* = 10 per group. **g** Sarcomere structures detected by α-actinin (red) in the grafts (green). Scale bars, 20 µm. **h** Sarcomere length in the grafts. *n* = 150 per group. **i** Cell boundaries identified by wheat germ agglutinin (WGA, red) in the grafts. Boxed regions are shown at twofold magnification. Scale bars, 50 µm. **j** Cell sectional area in the grafts. *n* = 150 per group. **(k)** Cell nucleus (DAPI, blue) in the βMHC^+^ grafts (red). Scale bars, 50 µm. **l** Cell number in the grafts (mm^2^). *n* = 8 per group. All data are presented as the mean ± SEM, the *P* values were determined with unpaired t tests (*P < 0.05, ****P < 0.0001)

To evaluate engrafted hiPSC-CMs, we performed histological and immunohistochemical analyses by harvesting rat hearts. Consistent with the outcome of in vivo BLI, the mean graft size of D56-CMs was significantly larger than that of D28-CMs at 12 weeks post-transplantation (*n* = 10 per group, Fig. 3a, b, Additional file 1: Fig. S8a), although the initial graft size at 1 week after transplantation was not significantly different (*n* = 5 per group, Additional file 1: Fig. S8b). There was no significant difference in the infarcted area between the D28-CM and D56-CM groups (Fig. 3c, d). Since hPSC-CMs matured in vivo after engraftment in rat hearts in our previous studies [17, 25], we compared the maturity of the engrafted D28-CMs and D56-CMs. Increased sarcomere length and cTnI expression as well as polarized distribution of pan-cadherin, markers of mature CMs, were observed in the D56-CM grafts compared with the D28-CM grafts (Fig. 3e–h, Additional file 1: Fig. S8c). Collectively, these data indicated that extended in vitro culture enhanced hiPSC-CM engraftment efficiency and maturation in vivo post-transplantation.

To explore the mechanism for improved enlargement of the D56-CM graft, we further analyzed graft cells at 12 weeks post-transplantation. Although no significant difference in cell size was observed (Fig. 3i, j), the number of cells in the D56-CM grafts was significantly higher than that in the D28-CM grafts (Fig. 3k, l), indicating that the D56-CM graft enlargement was not caused by hypertrophy. Then, we investigated hiPSC-CM proliferation after transplantation. The proliferation rates of D28-CMs and D56-CMs detected by Ki-67 at 1 week post-transplantation were 8.6 ± 0.8% and 8.7 ± 0.7%, respectively (Additional file 1: Fig. S8d-e). The slight increase in the fraction of Ki-67-positive cells in D56-CMs in vivo compared to the in vitro cell preparation (Fig. 1k) may suggest that D56-CMs re-entered the cell cycle in vivo, and there was no significant difference in proliferative cells detected by Ki-67 and phosphorylated histone H3 (PH3) between the D28-CM and D56-CM grafts at 4–8 weeks post-transplantation (Additional file 1: Fig. S9a-f). These data indicated that the difference in proliferation rates did not cause better graft enlargement by D56-CMs. By a TUNEL assay, the D56-CM grafts contained slightly fewer apoptotic cells than the D28-CM grafts (Additional file 1: Fig. S10a-c). In conjunction with the in vitro cell viability assay (Fig. 1l) and a TUNEL assay after hypoxia–reoxygenation (Fig. 1m), these results suggested that D56-CMs are resistant to stress conditions such as reactive oxygen species, which may in part contribute to better engraftment.

### D56-CM promoted angiogenesis in vivo and directly enhanced endothelial migration in vitro

**Fig. 4:**
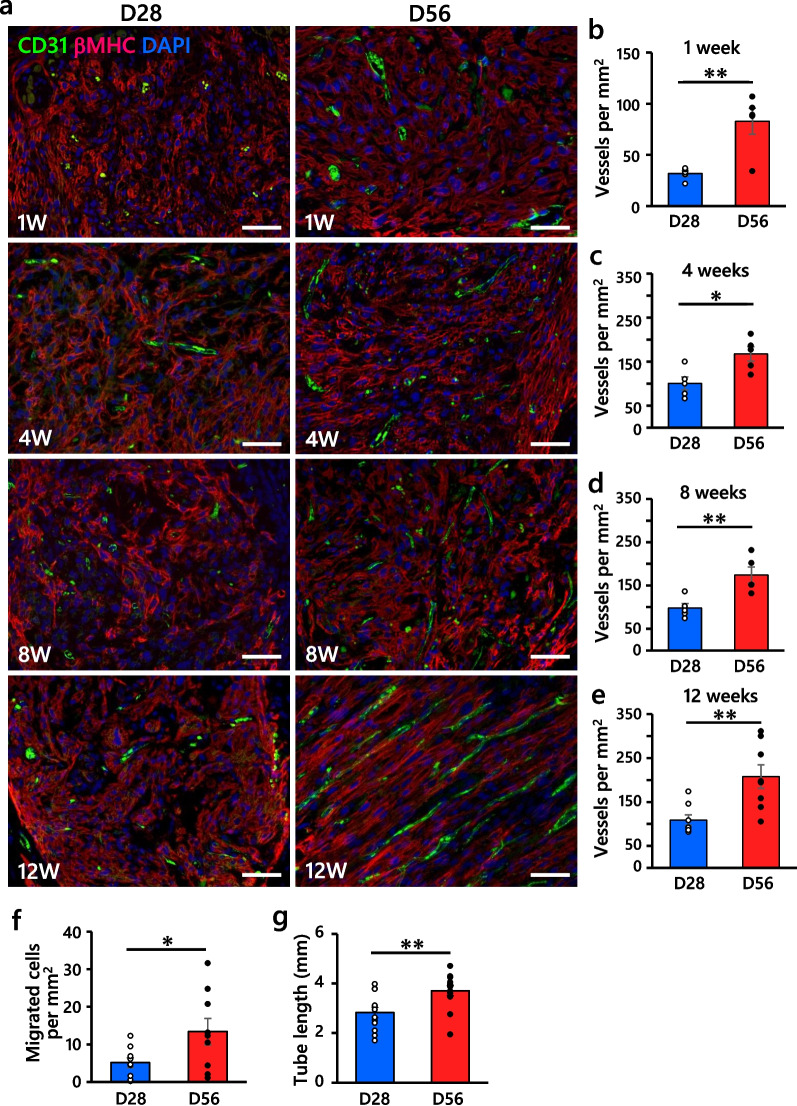
D56-CM promotes angiogenesis in vivo and in vitro. **a** Representative images of CD31^+^ microvessel (green) formation in βMHC^+^ grafts (red) at 1, 4, 8 and 12 weeks post-transplantation. Scale bars, 50 μm. **b**–**e** Quantification of microvessel formation in grafts over time post-transplantation. Five sites were randomly selected from each animal; 1, 4 and 8 weeks, *n* = 5 per group; 12 weeks, *n* = 8 per group. **f** Quantification of migrated HUVECs at 22 h after co-culture with hiPSC-CMs. *n* = 9 per group. **g** Quantification of tube lengths at 6 h after incubation of HUVECs with culture supernatants of hiPSC-CMs. *n* = 12 per group. All data are presented as the mean ± SEM, and the* P* values were determined with unpaired t tests (*P < 0.05, **P < 0.01)

Since hiPSC-CMs are transplanted into the ischemic area, neovascularization is essential for survival and proliferation after hiPSC-CM engraftment [43–45]. In this context, we investigated angiogenesis in the graft area as a cause of the graft size difference. Surprisingly, the number of CD31 (*PECAM1*)-positive microvessels in the D56-CM grafts was significantly increased as early as one week through 12 weeks post-transplantation compared to that in the D28-CM grafts (Fig. 4a-e). Microvessels were recognized as shown in Additional file 1: Fig. S11a. To determine whether the angiogenic potential was directly attributed to hiPSC-CMs, we performed a HUVEC migration assay after co-culturing with D28-CMs or D56-CMs in vitro (Additional file 1: Fig. S11b). D56-CMs significantly promoted the migration of HUVECs compared to D28-CMs (Fig. 4f, Additional file 1: Fig. S11c). D56-CMs also promoted HUVEC tube formation compared to D28-CMs (Fig. 4g, Additional file 1: Fig. S11d). To identify the factors responsible for increased cell migration and tube formation in D56-CMs, we performed human angiogenesis proteome profiler array analysis, but there was no significant difference in already-known angiogenic protein levels, including vascular endothelial growth factor A (*VEGFA*), fibroblast growth factor (*FGF2*) and angiopoietin 1 (*ANGPT1*), between the cell lysates of D28- and D56-hiPSC-CMs (Additional file 1: Fig. S12a). Consistently, no significant difference in the expression of the aforementioned angiogenic factors was measured by qRT-PCR (Additional file 1: Fig. S12b-d).

### Engrafted D56-CM-enriched genes related to blood vessel regulation

**Fig. 5:**
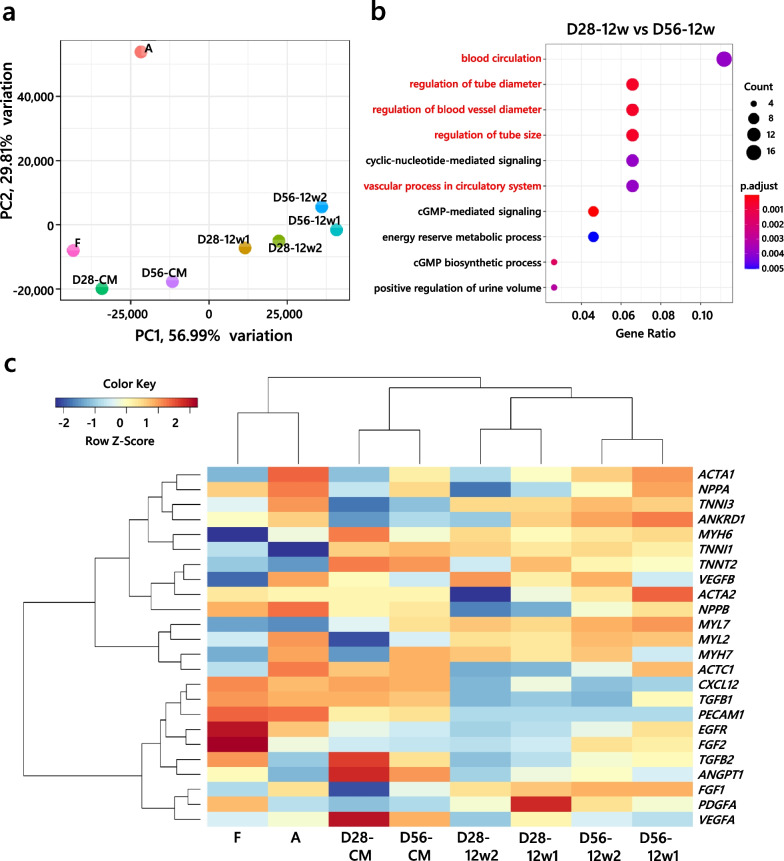
RNA-seq analysis of in vitro and in vivo hiPSC-CM samples. **a** Principal component analysis of RNA-seq data. **b** The top 10 enriched Gene Ontology terms in engrafted D56-CMs compared with D28-CMs at 12 weeks post-transplantation. Half of the top 10 (marked in red) are related to blood vessels. **c** Heatmap of selected sarcomere- and angiogenesis-related genes. D28-CM and D56-CM: in vitro samples; D28-12w1 and D28-12w2: in vivo engrafted D28-CMs at 12 weeks post-transplantation; D56-12w1 and D56-12w2: in vivo engrafted D56-CMs at 12 weeks post-transplantation; F and A: fetal and adult human heart samples as references

Next, we performed RNA-seq analysis to reveal the differences in transcriptomic profiles before and 12 weeks after transplantation. We obtained RNA samples from in vivo engrafted cells via a laser microdissection technique and distinguished human- and rat-derived reads to further analyze the human-derived reads [25]. Principal component analysis revealed that both D28-CMs and D56-CMs had expression levels close to the fetal human heart sample and that gradual maturation was observed under in vivo conditions, although no hiPSC-CMs reached the level of adult heart sample (Fig. 5a). At 12 weeks after cell engraftment, GO analysis revealed that D56-CM-enriched genes were associated with GO terms related to blood vessel regulation (Fig. 5b; Additional file 1: Table S3). Consistent with the qRT-PCR (Fig. 1d–f) and immunohistochemical analyses (Fig. 3e–h), mature sarcomere genes were upregulated in D56-CMs under in vitro and in vivo conditions, but the expression of known angiogenic factors did not differ between D56-CMs and D28-CMs (Fig. 5c), as shown in the human angiogenesis proteome profiler array analysis.

### Alpha-B crystallin (CRYAB) was identified as an angiogenic factor

**Fig. 6:**
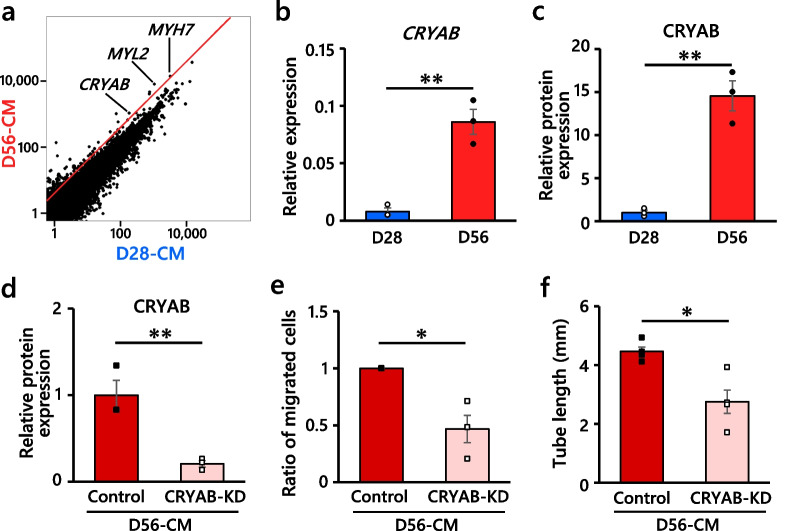
CRYAB was identified as an angiogenic factor of D56-CMs. **a** Scatter plot of RNA-seq data of D28-CMs and D56-CMs. The red line represents fourfold upregulation in D56-CMs compared with D28-CMs. **b** qRT-PCR analysis validated the upregulation of CRYAB in D56-CMs. The value for an adult heart sample was set to 1 as a reference. *n* = 3 per group. **c** Western blot analysis also confirmed the upregulation of CRYAB protein in D56-CMs compared with D28-CMs. GAPDH protein was used as a loading control. *n* = 3 per group. **d** Western blot analysis confirmed the KD of CRYAB (CRYAB-KD) after siRNA treatment in D56-CMs. As a control, non-targeting siRNA was used. *n* = 3 per group. **e** CRYAB-KD inhibited HUVEC migration by D56-CMs. The ratio of migrated cells in three independent experiments (*n* = 3–6 per experiment) is shown. **f** CRYAB-KD inhibited HUVEC tube lengths by D56-CMs. *n* = 4 per group. All data are the mean ± SEM, and the *P* values were determined with unpaired t tests (*P < 0.05, **P < 0.01)

Through further RNA-seq analysis, the expression of the small heat shock protein *CRYAB* was found to be upregulated in D56-CMs compared to D28-CMs (Fig. 6a; Additional file 1: Table S4), as validated by qRT-PCR (Fig. 6b, Additional file 1: Fig. S13a) and western blot analysis (Fig. 6c, Additional file 1: Fig. S13b). CRYAB was reported as a maturation-associated protein of hPSC-CMs [24]; in addition, this protein has proangiogenic properties in non-cardiac cells [46–48]. To elucidate the angiogenic effects of CRYAB, we transfected 10 nM small interfering RNA (siRNA) against *CRYAB* into D56-CMs 2–3 days prior to the HUVEC migration assay. siRNA-mediated knockdown of CRYAB was confirmed via western blot analysis (Fig. 6d, Additional file 1: Fig. S13c). Notably, HUVEC migration was significantly inhibited post-transfection with *CRYAB*-siRNA, whereas this effect was not observed with the non-targeting siRNA control (Fig. 6e). Tube formation was also inhibited by *CRYAB*-siRNA (Fig. 6f). Thus, we identified CRYAB, which is highly expressed in D56-CMs, as an angiogenic factor of hiPSC-CMs. Recently, CRYAB was reported to be secreted from CMs through exosomes [49]; thus, we measured CRYAB concentrations in the exosomes extracted from the culture supernatants by ELISA. The mean CRYAB concentration in the exosomes from D56-CMs was slightly higher than that from D28-CMs (Additional file 1: Fig. S13d), although the CRYAB concentrations in the culture supernatants of both D28-CMs and D56-CMs were undetectable.

**Fig. 7:**
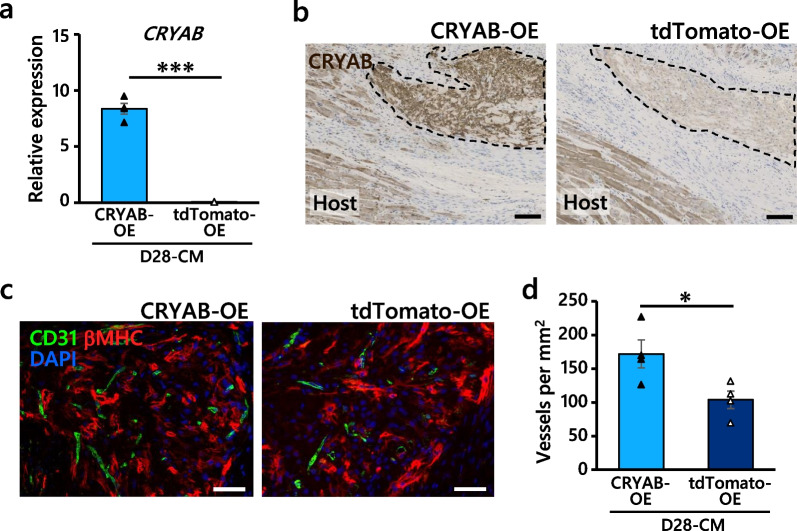
CRYAB-overexpressing D28-CMs enhanced angiogenesis in vivo*.* **a** qRT-PCR analysis validated AAV-mediated CRYAB overexpression (CRYAB-OE) 5 days after infection. An AAV vector carrying only tdTomato (tdTomato-OE) was used as a control. *n* = 4 per group. The value for an adult heart sample was set to 1 as a reference. **b** Immunostaining also confirmed significant upregulation of CRYAB in CRYAB-OE grafts (left) compared to tdTomato-OE grafts (right). Grafts are indicated by dotted lines. Scale bars, 100 µm. **c** Representative images of CD31^+^ microvessel (green) formation in βMHC^+^ grafts (red) at 4 weeks post-transplantation. Scale bars, 50 μm. **d** Quantification of microvessel formation in AAV-infected D28-CM grafts. Five sites were randomly selected from each animal. *n* = 4 per group. All data are the mean ± SEM, and the *P* values were determined with unpaired t tests (*P < 0.01, ***P < 0.001)

Furthermore, we infected D28-CMs with an adeno-associated virus (AAV) encoding the human *CRYAB* gene (Additional file 1: Fig. S14a) to investigate the angiogenic effects of *CRYAB* in hiPSC-CMs in vivo. Significant upregulation of CRYAB by the AAV-mediated *CRYAB* overexpression (CRYAB-OE) compared with the empty vector (tdTomato-OE) was validated via qRT-PCR and immunohistochemical analysis (Fig. 7a, b, Additional file 1: Fig. S14b). Cell viability was slightly higher in the CRYAB-OE D28-CMs than in the tdTomato-OE D28-CMs after the administration of 100 µM H_2_O_2_ (Additional file 1: Fig. S14c). Notably, AAV-mediated CRYAB overexpression enhanced angiogenesis in the D28-CM grafts 4 weeks after transplantation (Fig. 7c, d), although there was no significant difference in apoptosis in engrafted D28-CMs (Additional file 1: Fig. S14d). Collectively, these data indicated that CRYAB expressed in hiPSC-CMs enhanced endothelial cell migration and tube formation in vitro and angiogenesis in vivo*.*

## Discussion

hPSC-CMs exhibit a fetal-like immature phenotype, which has been established through structural, transcriptomic and proteomic analyses [22, 50]. In previous studies, the in vivo environment promoted hPSC-CM maturation compared with in vitro long-term culture [13, 17, 25]. Thus, we initially hypothesized that prolonged in vivo conditions, as opposed to extended in vitro culture, can promote hiPSC-CM maturation post-transplantation. Contrary to our hypothesis, however, extended in vitro culture of hiPSC-CMs could enlarge graft size and enhance cell maturation. The most striking finding of this study was the promotion of angiogenesis by an angiogenic factor, CRYAB*,* which is abundantly expressed in mature hiPSC-CMs. This effect was confirmed with in vitro endothelial cell migration and tube formation assays. We also confirmed that CRYAB overexpression can enhance angiogenesis in vivo in hiPSC-CM grafts.

Prior to commencing this study, we speculated that initial graft retention and cell proliferation are two major factors responsible for engraftment efficiency. Our in vivo BLI study using Akaluc-expressing hiPSCs demonstrated that there was no difference in the early-stage retention of D28-CMs and D56-CMs, indicating that extended culture of CMs has no effect on early cell retention or engraftment. Proliferative hiPSC-CMs have been shown to enhance engraftment after transplantation [6, 7]; however, in the current study, mature hiPSC-CMs showed a decreased proliferative capacity before transplantation, and ultimately, there was no difference in the in vivo proliferation of D28-CMs and D56-CMs detected by Ki-67 and PH3 staining. Therefore, the delayed enhancement of bioluminescence intensity in the D56-CM grafts was not due to differences in either initial retention or cell proliferation.

Next, we investigated angiogenesis, which is an important factor for graft enlargement [51]. Enhanced local vascularization and blood supply are thought to promote survival, proliferation and functional improvement of grafted cells [52]. Co-transplantation with endothelial cells [4], microvessels [43] or epicardial cells [32] was reported to promote hPSC-CM graft enlargement. Our study revealed that neovascularization was notably observed in the graft area at early time points after D56-CM transplantation, even though we did not co-transplant non-CM populations. Accordingly, the delayed enhancement of the bioluminescence increase was attributed to the enhanced distribution of the luminescent substrate as a result of angiogenesis in the grafts [53, 54]. CMs secrete several angiogenic factors, such as acidic and basic FGFs [55] and VEGF [56]. Although no significant upregulation of 55 known angiogenic proteins was detected in D56-CMs compared with D28-CMs through human angiogenesis proteome profiler array analysis, we identified a small heat shock protein, CRYAB, as an angiogenic factor that was upregulated in D56-CMs, as shown by RNA-seq, qRT-PCR and western blot analyses.

Small heat shock proteins are the key components of the cellular protein control system and act as chaperones for unfolded proteins. Other binding targets of small heat shock proteins are proteins that are involved in various cellular processes, such as signal transduction, transcription, translation, autophagy, apoptosis and cytoskeleton stabilization [57]. CRYAB is a component of the sarcomere structure in CMs that interacts with titin [58] and desmin [59]. A proteomic study revealed that the expression of CRYAB was upregulated in 59-day-old CMs compared to 30-day-old CMs [24]. In our current study, *CRYAB*-siRNA inhibited endothelial cell migration in vitro. Moreover, CRYAB overexpression enhanced angiogenesis in the D28-CM grafts in vivo. CRYAB has been reported to regulate angiogenesis in retinal pigment epithelial [60], breast cancer [61] and gastric cancer cells [62]; however, to our knowledge, no reports have indicated that CRYAB expression in mature CMs promotes angiogenesis both in vitro and in vivo. CRYAB can regulate angiogenesis through the VEGF signaling pathway in other cell types [60, 61]. We detected slightly higher CRYAB concentrations in the exosomes isolated from the culture supernatants of D56-CMs; therefore, we thought that CRYAB affected endothelial cells directly through exosomes. However, CRYAB may regulate other angiogenic factors intracellularly; thus, further study is needed to reveal the mechanism by which CRYAB in hiPSC-CMs promotes angiogenesis. In ischemic rat hearts, CRYAB modulated CM apoptosis, which blocked mitochondrial outer membrane channels [63]. In our study, we found that upregulation of CRYAB in extended cultured hiPSC-CMs was concomitant with increased angiogenesis as well as anti-apoptotic effects. One might think that the anti-apoptotic effect was related to angiogenesis; however, this effect was observed only in in vitro experiments, suggesting an independent mechanism from angiogenesis.

Despite significantly better engraftment with more mature D56-CMs, we did not see any reduction in the infarcted area compared to that observed for D28-CMs. Although many animal studies have shown that graft size is inversely correlated with the infarcted area [4, 6, 7, 43, 44], increased hPSC-CM engraftment did not change the infarct size in a rat study [32]. Thus, better engraftment may not always confer substantial additional protection against scar formation. In addition, mature D56-CMs showed cardiac functional improvement measured by echocardiography, but no additional benefit was observed compared to immature D28-CMs. The mechanical benefits from hPSC-CM grafts are believed to be derived from two distinct mechanisms: direct force generation from electrically integrated new CMs [64] and indirect paracrine mechanisms [65]. Given the extremely fast beating rate of the recipient rat heart, this model is not ideal for detecting subtle changes by mature hiPSC-CMs. It would be better to compare mechanical restoration between recipients of immature and mature hPSC-CMs in slower heart rate, large animal models, which is beyond the scope of the current study.

Recently, transplantation of artificially matured hPSC-CMs into guinea pig hearts was shown to promote the development of structural maturation, improve host–graft electromechanical integration, decrease proarrhythmic behavior and exert beneficial effects on contractile function [22]. Although improved engraftment of the matured CMs was observed, there was no increased microvessel formation in the matured hPSC-CM grafts. In contrast, mature compact ventricular hPSC-CMs did not increase engraftment after transplantation in injured rat hearts [21]. The different maturation protocols and possibly different maturation statuses might result in opposite results regarding graft enlargement. In this context, it is crucial to identify a marker suggesting an optimal maturation status for better engraftment. Here, we reveal that CRYAB is also useful as an indicator of angiogenic mature CMs, leading to reasonably good engraftment. A single-cell RNA-seq study of adult human hearts revealed that *CRYAB* expression was upregulated in less than 10% of the population of ventricular CMs, which can tolerate stress conditions and high workloads [66]. Therefore, further studies are necessary to investigate the regulation of CRYAB expression in hiPSC-CMs and the detailed mechanisms of angiogenesis and graft enlargement for the development of efficient and effective cardiac regenerative therapy.

## Conclusion

Extended culture of hiPSC-CMs promoted remuscularization with enhanced angiogenesis post-transplantation in rat infarcted hearts. We identified an angiogenic factor, CRYAB*,* responsible for this outcome in extended cultured hiPSC-CMs. This novel angiogenic factor-based therapy may elucidated the optimal timing for cell harvest and maturation status of hPSC-CMs for cardiac regeneration, bringing this therapy closer to standard practice.

### Supplementary Information

**Additional file1**. **Fig S1.** Generation of the Akaluc-expressing hiPSC line. **Fig S2.** Flow cytometric analysis. **Fig S3.** qRT-PCR analysis. **Fig S4.** Immunocytochemistry. **Fig S5.** Comparison of Luc2- and Akaluc-expressing hiPSCs. **Fig S6.** Echocardiography. **Fig S7.** BLI. **Fig S8.** Assessment of the graft size and composition of hiPSC-CMs at 1 and 12 weeks post-transplantation. **Fig S9.** Cell proliferation in grafts at 4 and 8 weeks post-transplantation. **Fig S10.** Apoptosis in grafts at 4 and 8 weeks post-transplantation. **Fig S11.** Microvessels in grafts and in vitro assays for assessing angiogenesis by hiPSC-CMs. **Fig S12.** Angiogenesis profiler array and qRT-PCR. **Fig S13.** Expression of CRYAB in hiPSC-CMs. **Fig S14.** AAV-mediated CRYAB overexpression. **Table S1.** Primer sequences for genomic PCR and qRT-PCR in this study. **Table S2.** Antibodies used for immunohistochemistry. **Table S3.** Top 10 gene ontology (GO) terms enriched in D56-CM compared with D28-CM at 12 weeks after transplantation. **Table S4.** Top 20 genes with TPM-normalized counts upregulated by more than 4-fold in D56-CMs compared with D28-CMs before transplantation.

## Data Availability

RNA-seq data were deposited in the NCBI Gene Expression Omnibus (GEO series accession number GSE164919; https://www.ncbi.nlm.nih.gov/geo/query/acc.cgi?acc=GSE164919).

## Abbreviations

AAV: Adeno-associated virus
AAVS1: Adeno-associated virus integration site 1
ANOVA: Analysis of variance
BLI: Bioluminescence imaging
CRISPR/Cas9: Clustered regularly interspaced short palindromic repeats (CRISPR)-associated nuclease 9
CMs: Cardiomyocytes
CRYAB: Alpha-B crystallin
cTnI: Cardiac troponin I
cTnT: Cardiac troponin T
DAPI: 4′,6-Diamidino-2-phenylindole
D28-CMs: hiPSC-CMs collected on day 28 after differentiation
D56-CMs: hiPSC-CMs collected on day 56 after differentiation
EdU: 5-Ethynyl-2′-deoxyuridine
ELISA: Enzyme-linked immunosorbent assay
GAPDH: Glyceraldehyde-3-phosphate dehydrogenase
GFP: Green fluorescent protein
hiPSC-CMs: Human induced pluripotent stem cell-derived cardiomyocytes
HUVEC: Human umbilical vein endothelial cell
KD: Knockdown
OE: Overexpression
PH3: Phosphorylated histone H3
qRT-PCR: Quantitative reverse transcription polymerase chain reaction
RNA-seq: RNA sequencing
siRNA: Small interfering RNA
TUNEL: Terminal deoxynucleotidyl transferase dUTP nick end labeling
βMHC: Beta myosin heavy chain
