# Supplementary material for: Mature human induced pluripotent stem cell-derived cardiomyocytes promote angiogenesis through alpha-B crystallin
Source: Stem Cell Res Ther. 2023 Sep 7;14:240. doi: 10.1186/s13287-023-03468-4 (PMC10486094; doi:10.1186/s13287-023-03468-4)
Supplement: Supplementary file 1 — Additional file1. Fig S1. Generation of the Akaluc-expressing hiPSC line. Fig S2. Flow cytometric analysis. Fig S3. qRT-PCR analysis. Fig S4. Immunocytochemistry. Fig S5. Comparison of Luc2- and Akaluc-expressing hiPSCs. Fig S6. Echocardiography. Fig S7. BLI. Fig S8. Assessment of the graft size and composition of hiPSC-CMs at 1 and 12 weeks post-transplantation. Fig S9. Cell proliferation in grafts at 4 and 8 weeks post-transplantation. Fig S10. Apoptosis in grafts at 4 and 8 weeks post-transplantation. Fig S11. Microvessels in grafts and in vitro assays for assessing angiogenesis by hiPSC-CMs. Fig S12. Angiogenesis profiler array and qRT-PCR. Fig S13. Expression of CRYAB in hiPSC-CMs. Fig S14. AAV-mediated CRYAB overexpression. Table S1. Primer sequences for genomic PCR and qRT-PCR in this study. Table S2. Antibodies used for immunohistochemistry. Table S3. Top 10 gene ontology (GO) terms enriched in D56-CM compared with D28-CM at 12 weeks after transplantation. Table S4. Top 20 genes with TPM-normalized counts upregulated by more than 4-fold in D56-CMs compared with D28-CMs before transplantation. [file 13287_2023_3468_MOESM1_ESM.docx]

**Supplementary Figures**

**Fig S1. Generation of the Akaluc-expressing hiPSC line**

**(a)** Schematic diagram of the CRISPR/Cas9-mediated knock-in strategy at the *AAVS1* locus. The vertical arrow indicates the single guide RNA (sgRNA) targeting site. Horizontal arrows are PCR primers for assaying the *AAVS1* locus, the Akaluc sequence and the puromycin selection cassette.

**(b, c)** Genomic PCR analysis of correctly targeted clones. PCR products of heterozygously targeted clones before **(b)** and after **(c)** the removal of the selection cassette by transfection of the pCAG-iCre plasmid.

**Fig S2. Flow cytometric analysis**

**(a)** Representative flow cytometric analyses of cTnT^+^ and EdU^+^ proliferating hiPSC-CMs derived from 253G1 hiPSCs.

**(b)** Representative flow cytometric analysis of D56-CMs derived from 253G1 hiPSCs. Isotype control (left), EdU staining (middle), and cTnT + EdU double staining (right) in the same sample.

**Fig S3. qRT-PCR analysis**

Relative mRNA expression of mature sarcomere **(a-c)**, immature sarcomere **(e)**, hypertrophy **(h, i)**, and calcium handling **(j, k)**-related genes in D28-CMs (blue) and D56-CMs (red).

**(d, f, g)** Ratio of mature/immature sarcomere gene expression in D28-CMs (blue) and D56-CMs (red).

The value for an adult heart sample was set to 1 as a reference. n = 3 each. All data are presented as the mean ± SEM, and the *P* values were determined with unpaired t tests (**P* < 0.05, ***P* < 0.01).

**Fig S4. Immunocytochemistry**

**(a, c)** Representative images of mature CMs derived from 201B7 **(a)** and 610B1 **(c)** hiPSCs detected by cTnI and cTnT immunostaining. Scale bars, 100 µm.

**(b, d)** Percentage of mature CMs derived from 201B7 **(b)** and 610B1 **(d)** hiPSCs. n = 3 each.

All data are presented as the mean ± SEM, and the *P* values were determined with unpaired t tests (**P* < 0.05).

**(e)** Representative images of apoptotic CMs after hypoxia-reoxygenation shown in **Fig. 1m**. Scale bars, 100 µm.

**Fig S5. Comparison of Luc2- and Akaluc-expressing hiPSCs**

**(a)** *In vivo* BLI of subcutaneously transplanted 5×10^5^ undifferentiated Luc2- (left) and Akaluc-expressing (right) hiPSCs. **(b)** Quantification of the bioluminescence intensity obtained from **(a)**.

**Fig S6. Echocardiography**

**(a)** Representative images of M-mode echocardiography at 12 weeks post-transplantation of hiPSC-CMs (D28, D56) and PBS-treated control groups.

Left ventricular ejection fraction **(b)**, end-diastolic dimensions (LVEDd) **(d)** and end-systolic dimensions (LVEDs) **(e)** before and after transplantation of hiPSC-CMs (D28, D56) and PBS-treated control groups. n=5 per group. ***P* < 0.01 vs. the value pre-transplantation within the group, one-way ANOVA with Tukey’s multiple comparisons test.

**(c)** Change in ejection fraction from pre-transplantation to 12 weeks post-transplantation. n=5 per group. ***P* < 0.01 vs. control group, one-way ANOVA with Tukey’s multiple comparisons test.

**Fig S7. BLI**

**(a)** Representative images of bioluminescence signals (ph/s; photons per sec) of Akaluc-expressing D28-CMs (upper 3 lanes) and D56-CMs (lower 3 lanes).

**(b)** Correlation of bioluminescence intensity with cell quantity. Pearson’s correlation coefficient test, *P* = 0.9999 in both groups, n = 3 per group.

**(c)** Time-course analysis of individual bioluminescence intensity after transplantation shown in **Fig. 2e**. At 8 and 12 weeks post-transplantation, the D56-CM-treated group (n=6, red circle) showed a higher signal intensity than the D28-CM-treated group (n=5, blue square), except for one D28-CM-treated rat at week 12.

**Fig S8. Assessment of the graft size and composition of hiPSC-CMs at 1 and 12 weeks post-transplantation**

**(a)** Quantification of the graft area detected by GFP staining at 12 weeks post transplantation. The D56-CM graft was larger than the D28-CM graft. n = 10 per group.

**(b)** There was no significant difference in the initial graft size at 1 week after transplantation between the D28-CM- and D56-CM-treated groups. n = 5 per group.

**(c)** Representative images of adherens junctions identified by pan-cadherin (red) in GFP^+^ grafts at 12 weeks post-transplantation. Adherens junctions were polarized to the end of the cells (white arrowheads). Scale bars, 20 μm.

**(d)** Representative images of proliferating CMs detected by Ki-67 (green) and Ku80 (human nuclei, blue) in βMHC^+^ grafts (red) at 1 week post-transplantation. Scale bars, 50 μm.

**(e)** There was no significant difference in proliferating CMs at 1 week post-transplantation. n = 5 per group.

All data are the mean ± SEM, and *P* value was determined with the unpaired t test (**P*<0.05).

**Fig S9. Cell proliferation in grafts at 4 and 8 weeks post-transplantation**

**(a)** Representative images of proliferating cells detected by Ki-67 (green) staining in βMHC^+^ grafts (red) at 4 weeks (upper panels) and 8 weeks (lower panels) post-transplantation. Scale bars, 50 μm.

**(b-c)** Quantification of proliferating cells detected by Ki-67 staining in the engrafted area at **(b)** 4 weeks and **(c)** 8 weeks post-transplantation. n = 5 per group.

**(d)** Representative images of mitotic cells visualized by PH3 (green) in βMHC^+^ grafts (red) at 4 weeks (upper panels) and 8 weeks (lower panels) post-transplantation. Nuclei were stained with DAPI. Scale bars, 20 μm.

**(e-f)** Quantification of cells undergoing mitosis detected by PH3 in the engrafted area at **(e)** 4 weeks and **(f)** 8 weeks post-transplantation. n = 5 per group.

All data are the mean ± SEM.

**Fig S10. Apoptosis in grafts at 4 and 8 weeks post-transplantation**

**(a)** Representative images of cells undergoing apoptosis detected by TUNEL (red), Ku80 (human nuclei, blue), GFP (green) and α-actinin (white) at 4 weeks (upper panels) and 8 weeks (lower panels) post-transplantation. Scale bars, 20 μm.

**(b-c)** Quantification of apoptotic (TUNEL^+^) hiPSC-CMs (Ku80^+^, GFP^+^, α-actinin^+^) at **(b)** 4 weeks and **(c)** 8 weeks post-transplantation. n = 5 per group.

All data are the mean ± SEM, and *P* values were determined with the unpaired t test (**P*<0.05, ***P*<0.01).

**Fig S11. Microvessels in grafts and *in vitro* assays for assessing angiogenesis by hiPSC-CMs**

**(a)** An example image in **Fig. 4a** (1 week after transplantation of D56-CMs) showing how to recognize blood vessels. We counted CD31^+^ lumen structures (white arrowheads) as blood vessels but excluded non-specific stains (white arrows). Scale bar, 20 μm.

**(b)** Schematic representation of the HUVEC migration assay.

**(c)** Representative images of migrated HUVECs after co-culturing with hiPSC-CMs or negative control (Neg; without hiPSC-CMs). Arrowheads indicate migrated cells detected by crystal violet staining. The surrounding circles represent the pores of the membrane. Scale bar, 100 μm.

**(d)** Representative images showing tube formation at 6 h after incubation of HUVECs with culture supernatants of D28-CMs or D56-CMs. Scale bar, 200 μm.

**Fig S12. Angiogenesis profiler array and qRT-PCR**

**(a)** Angiogenesis-related protein expression detected in 253G1 hiPSC-CMs on days 28 (blue) and 56 (red) by human angiogenesis profiler array analysis. Reference spot = 1, n = 3 per group.

**(b-d)** Relative mRNA expression of angiogenesis-related genes in D28-CMs (blue) and D56-CMs (red). The value for an adult heart sample was set to 1 as a reference. n = 3 each.

All data are presented as the mean ± SEM.

**Fig S13. Expression of CRYAB in hiPSC-CMs**

**(a)** qRT-PCR analysis validated the upregulation of *CRYAB* in 201B7 and 610B1 D56-CMs. n = 3 per group. The *P* value was determined with an unpaired t test (***P*<0.01). The value for an adult heart sample was set to 1 as a reference.

**(b)** Western blot image showing CRYAB protein levels in hiPSC-CMs shown in **Fig. 6c**. GAPDH served as a loading control.

**(c)** Western blot image showing CRYAB KD by siRNA treatments in D56-CMs shown in **Fig. 6d**.

**(d)** Quantification of CRYAB concentrations in the exosomes isolated from culture supernatants of hiPSC-CMs measured by ELISA. n = 3 per group. The *P* value was determined with the Mann-Whitney U test (**P* < 0.05).

All data are the mean ± SEM.

**Fig S14. AAV-mediated CRYAB overexpression**

**(a)** Fluorescence images of tdTomato expression at day 5 post-infection with AAV6-CMV-CRYAB-P2A-tdTomato (CRYAB, middle) or AAV6-CMV-tdTomato (tdTomato, right). The negative control (Neg: noninfected D28-CMs, left) showed no expression. Scale bars, 1 mm.

**(b)** Immunostaining of GFP (indicated by dotted lines) detected the grafts of CRYAB-overexpressing (CRYAB-OE, left) or tdTomato-overexpressing (tdTomato-OE, right) D28-CMs in serial sections of **Fig. 7b**. Scale bars, 100 µm.

**(c)** Quantification of cardiomyocyte viability after treatment with H_2_O_2_ (100 µM) for 2 h. Each value was compared with that of the H_2_O_2_ nontreated group. n = 15 per group.

**(d)** Quantification of cells undergoing apoptosis of transplanted hiPSC-CMs at 4 weeks post-transplantation. n = 4 per group.

All data are presented as the mean ± SEM, and the *P* value was determined with the Mann-Whitney U test (**P* < 0.05).

**Supplementary Tables**

**Table S1. Primer sequences for genomic PCR and qRT-PCR in this study**

| Genomic PCR | Forward primer | Reverse primer |
| --- | --- | --- |
| P1 | CTCTTCTCTGTTCAGCCCTAAGAATC |  |
| P2 | GGAGGAGCTATTCTTGCGCAGC |  |
| P3 |  | CGCGCGTGAGGAAGAGTTCTTG |
| P4 |  | TGATGGATATCTGCAGAATTCTTACACGGCGATCT |
| P5 |  | CATAGCTCAGTCTGGTCTATCTGCC |
| qRT-PCR | Forward primer | Reverse primer |
| *TNNT2* | TTCACCAAAGATCTGCTCCTCGCT | TTATTACTGGTGTGGAGTGGGTGTGG |
| *MYH6* | CTCAAGCTCATGGCCACTCT | GCCTCCTTTGCTTTTACCACT |
| *MYH7* | ACAAGCTGCAGCTAAAGGTC | TCAAGATGTGGCAAAGCTAC |
| *TNNI1* | CTCTTCAGCAAGAGTTTGCG | CAGCTCCACGAGGACTGAAC |
| *TNNI3* | CAGTAGGCAGGAAGGCTCAG | CCTCAAGCAGGTGAAGAAGG |
| *MYL7* | GTCTTCCTCACGCTCTTTGG | CCACCTCAGCTGGAGAGAAC |
| *MYL2* | TTGGGCGAGTGAACGTGAAAA | CCGAACGTAATCAGCCTTCAG |
| *GAPDH* | ATGGAAATCCCATCACCATCTT | CGCCCCACTTGATTTTGG |
| *ATP2A2* | CATGACAACCCACTGAGAAGAGAA | CGAAGGTCAGATTGGTCTCATATTT |
| *RYR2* | CTGCGCCATTCCTATAGTGG | AGTTGAAGACCGGGAGGTG |
| *NPPA* | TCCAACGCAGACCTGATGGA | GGGCACGACCTCATCTTCTA |
| *NPPB* | TGGAAACGTCCGGGTTACAG | CTTCCAGACACCTGTGGGAC |
| *CRYAB* | CACCCAGCTGGTTTGACACT | TTCCTCTGGGGAGAAGTGCT |

**Table S2. Antibodies used for immunohistochemistry**

| **Antibody** | **Species** | **Dilution** | **Manufacturer** | **Cat# or Clone** |
| --- | --- | --- | --- | --- |
| Green Fluorescent Protein | Rabbit | 1: 2000 | Novus | NB600-308 |
| Green Fluorescent Protein | Goat | 1: 500 | Novus | NB100-1770 |
| Beta-Myosin Heavy Chain | Mouse | 1: 20 | DSHB | A4.951 |
| Nucleolin (human) | Mouse | 1: 500 | Abcam | ab136649 |
| Cardiac Troponin I | Rabbit | 1: 200 | Abcam | ab47003 |
| Cardiac Troponin T | Mouse | 1: 200 | Thermo Fisher | 13-11 |
| Sarcomeric Alpha Actinin | Mouse | 1: 200 | Abcam | ab9465 |
| Pan Cadherin | Mouse | 1: 200 | Abcam | ab6528 |
| Ki-67 | Mouse | 1: 200 | Dako | M7240 |
| Ki-67 | Rabbit | 1: 200 | Abcam | ab16667 |
| Phospho-Histone H3 (Ser10) | Rabbit | 1: 200 | Cell Signaling | 9701 |
| Ku80 | Rabbit | 1: 100 | Abcam | ab80592 |
| CD31/PECAM-1 | Rabbit | 1: 100 | Novus | NB100-2284 |
| Alpha-B Crystallin | Mouse | 1: 200 | Enzo Life Sciences | ADI-SPA-222 |
| Wheat Germ Agglutinin, Alexa Fluor 555 conjugate | - | 1: 500 | Thermo Fisher | W32464 |
| Goat anti-Rabbit IgG (HRP polymer) | Goat | 1:1 | Abcam | ab214880 |
| Goat anti-Mouse IgG (HRP polymer) | Goat | 1:1 | Abcam | ab214879 |
| Goat anti-Rabbit IgG (Alexa Fluor 488) | Goat | 1: 200 | Thermo Fisher | A32731 |
| Donkey anti-Goat IgG (Alexa Fluor 488) | Donkey | 1: 200 | Thermo Fisher | A11055 |
| Goat anti-Mouse IgG (Alexa Fluor 555) | Goat | 1: 200 | Thermo Fisher | A32727 |
| Donkey anti-Mouse IgG (Alexa Fluor 546) | Donkey | 1: 200 | Thermo Fisher | A10036 |
| Donkey anti-Rabbit IgG (Alexa Fluor 647) | Donkey | 1: 200 | Thermo Fisher | A31573 |
| Donkey anti-Goat IgG (Alexa Fluor 350) | Donkey | 1: 200 | Thermo Fisher | A21081 |

**Table S3. Top 10 Gene Ontology (GO) terms enriched in D56-CM compared with D28-CM at 12 weeks after transplantation**

| GO ID | Description | Gene Ratio | p.adjust | Gene ID | Count |
| --- | --- | --- | --- | --- | --- |
| GO:0019934 | cGMP-mediated signaling | 7/152 | 1.39E-05 | *NPPB/NPPA/APOE/ADORA2B/NPR1/GUCY1B1/EDNRB* | 7 |
| GO:0035296 | regulation of tube diameter | 10/152 | 0.000465 | *NPPB/NPPA/EGFR/APOE/ADORA2B/SLC6A4/NPR1/ACTA2/EDNRB/KEL* | 10 |
| GO:0097746 | regulation of blood vessel diameter | 10/152 | 0.000465 | *NPPB/NPPA/EGFR/APOE/ADORA2B/SLC6A4/NPR1/ACTA2/EDNRB/KEL* | 10 |
| GO:0035150 | regulation of tube size | 10/152 | 0.000465 | *NPPB/NPPA/EGFR/APOE/ADORA2B/SLC6A4/NPR1/ACTA2/EDNRB/KEL* | 10 |
| GO:0006182 | cGMP biosynthetic process | 4/152 | 0.001587 | *NPPB/NPPA/NPR1/GUCY1B1* | 4 |
| GO:0035810 | positive regulation of urine volume | 4/152 | 0.003065 | *NPPB/NPR3/NPR1/EDNRB* | 4 |
| GO:0019935 | cyclic-nucleotide-mediated signaling | 10/152 | 0.003861 | *NPPB/NPPA/ADGRL3/APOE/ADORA2B/NPR1/GUCY1B1/RAPGEF3/EDNRB/MRAP2* | 10 |
| GO:0003018 | vascular process in circulatory system | 10/152 | 0.003861 | *NPPB/NPPA/EGFR/APOE/ADORA2B/SLC6A4/NPR1/ACTA2/EDNRB/KEL* | 10 |
| GO:0008015 | blood circulation | 17/152 | 0.003861 | *NPPB/NPR3/SGCG/NPPA/EGFR/TGFB2/APOE/ADORA2B/SLC6A4/NPR1/RNPEP/GUCY1B1/ACTA2/EDNRB/SCN4B/KCNK1/KEL* | 17 |
| GO:0006112 | energy reserve metabolic process | 7/152 | 0.00501 | *PGM2/KHK/PPP1R3C/MYC/PYGL/MRAP2/LEPR* | 7 |

**Table S4. Top 20 genes with TPM-normalized counts upregulated by more than 4-fold in D56-CMs compared with D28-CMs before transplantation**

| Gene ID | D28-CM | D56-CM | Fold Change |
| --- | --- | --- | --- |
| *MYH7* | 3231.836 | 13532.99 | 4.1874 |
| *MYL2* | 1074.759 | 7873.782 | 7.326089 |
| *NPPA* | 768.0746 | 4563.269 | 5.941179 |
| *ACTA1* | 753.8836 | 4014.779 | 5.325463 |
| *CRYAB* | 185.8436 | 1057.955 | 5.692714 |
| *TXNIP* | 75.67903 | 996.8405 | 13.17195 |
| *G0S2* | 85.89037 | 572.9574 | 6.6708 |
| *HSPB6* | 50.13312 | 289.5446 | 5.775516 |
| *TUBA4A* | 22.03583 | 140.6149 | 6.381192 |
| *MYL1* | 1.16394 | 135.694 | 116.5817 |
| *MSS51* | 11.78399 | 102.9208 | 8.733951 |
| *ARRDC4* | 17.00306 | 90.35343 | 5.313952 |
| *LINC01611* | 15.10058 | 75.82827 | 5.021546 |
| *BRINP3* | 14.98957 | 74.17032 | 4.948129 |
| *CASQ2* | 16.09433 | 68.24824 | 4.240514 |
| *SYNDIG1* | 12.85089 | 61.90576 | 4.817235 |
| *FGF12* | 1.285279 | 54.99318 | 42.78696 |
| *FRY* | 10.97212 | 54.11564 | 4.932106 |
| *CAV3* | 3.057573 | 42.71297 | 13.96957 |
| *CYP2J2* | 8.046569 | 41.33863 | 5.137423 |

TPM: transcripts per million.
